# Fractures Treated with Starched Bandages

**Published:** 1838

**Authors:** M. Velpeau


### Art. II.—Fractures treated with starched bandages.

M. Velpeau, lately read a notice of his mode of treatment, before the Royal Academy of Sciences. He immediately reduces the fracture in all cases, even when compound—the limb is then surrounded with a roller bandage, from the toes or fingers, to the proximal extremity—the pressure is merely sufficient to maintain the fragments in their proper direction; instead of using splints and pads, the envelope is made rigid by the application of starch prepared with water, after the plan of M. Seutin, of Brussels, though he uses a double bandage, pads, and pasteboard splints.—The whole apparatus dries in 2 or 4 days, when the limb and bandage are so glued together that displacement is impossible; compression is every where equalized, the tissues are sustained, and there is no pain. Patients can move in bed as if there were a simple bruise of the limb, instead of being forced to lie motionless for six or eight weeks. They may sit up using a high chair, so as to bend the thigh, but moderately—and they can walk with crutches, having the foot supported by a stirrup from their neck.—*Translated from the Archives Generales de Medicine.*

